# Maximizing the impact of community-based practitioners in the quest for universal health coverage

**DOI:** 10.2471/BLT.15.162198

**Published:** 2015-09-01

**Authors:** James Campbell, Kesetebirhan Admasu, Agnes Soucat, Sheila Tlou

**Affiliations:** a Global Health Workforce Alliance, World Health Organization, Avenue Appia 20, 1211 Geneva 27, Switzerland.; b Minister of Health, Federal Ministry of Health, Addis Ababa, Ethiopia.; c The World Bank, Washington, United States of America.; d Joint United Nations Programme on HIV/AIDS (UNAIDS), Johannesburg, South Africa.

The last decade has highlighted major gaps in the availability, accessibility, acceptability and quality of the health workforce in many countries.^1^ The quantity, skills and geographic distribution of the health workforce have long been recognized as factors that limit population health outcomes and progress towards the related Millennium Development Goals.^2^ Similarly, the even more ambitious health targets included in the Sustainable Development Goals – scheduled for adoption by the United Nations General Assembly later this month – may be undermined by the same factors.

Recognizing this reality, there have been calls for a paradigm shift in health workforce development efforts, moving towards a more diverse range of skills supporting primary health care.^3^ This has catalysed growing interest in, and attention to, the potential of various types of community-based practitioners in expanding access to essential health services, particularly in under-served communities in low- and middle-income countries.^4^ Several studies have demonstrated the potential of various types of community-based practitioner in delivering a range of health services.^5^^–^^7^ The new analysis by McPake and colleagues^8^ in this issue of the *Bulletin* adds an important dimension to this debate, by providing an empirical foundation to the argument. McPake et al. report that investment in these types of health workers can be a cost-effective approach, in certain contexts and under certain circumstances.

However, it is critical to take a broader health system perspective. As noted by the authors of this new analysis, the terms “frontline health workers” and “community health workers” are often used in a non-specific way and can confuse the evidence base. The term “frontline” is not a classification recognized by the World Health Organization (WHO) or the International Labour Organization (ILO). Even the official classification of community health workers can refer to a diverse typology of lay and educated, formal and informal, regulated and unregulated, paid and unpaid health workers. Different policies relating to individual cadres, their scope of practice, education and relation to the health system undermine efforts to strengthen service delivery at community level.^9^ Large-scale studies that have analysed the policy and health systems features of community-based practitioner programmes have identified major gaps relating to the inclusion of these cadres in national health systems and in the adoption of appropriate education, deployment, performance management and retention strategies.^10^

Fortunately, the elements needed for successful integration of community-based practitioners in health systems have been mapped.^11^ An extensive, global consultation on evidence relevant to the attainment of universal health coverage has been completed^12^ and the results translated into a draft of WHO’s *Global strategy on human resources for health: workforce 2030*.^13^ After consultations in all WHO Regions, the final version of this strategy will be considered by the World Health Assembly in 2016. The global strategy recognizes the potential of involving community-based, mid-level and advanced practitioners as part of a multi-disciplinary health workforce that offers people-centred, integrated primary health services.

Translating this vision into reality and leveraging the potential efficiency gains that McPake et al. have identified requires, among other things, that: (i) national policy-makers move towards the full integration of community-based practitioners in public health strategies, allowing these cadres to benefit from formal employment, education, health system support, regulation, supervision, remuneration and career advancement opportunities; (ii) development partners and funding agencies see the value of investing in these cadres and contribute to the capital and recurrent costs incurred when expanding this workforce; (iii) normative agencies such as WHO and ILO address the evidence and classification gaps by developing more precise definitions and categories for these cadres. 

To make the most of the investment opportunities that community-based, mid-level and advanced practitioners represent, policy-makers need to jointly support this agenda. Guidelines on the role, education and integration of community-based practitioners are being prepared by WHO for publication in 2017. These guidelines will provide governments and development partners with evidence-based recommendations on community-based practitioners, including potential returns on investment. These guidelines are intended to support universal health coverage and the achievement of the Sustainable Development Goals.
